# Mycotoxins in Cattle Feed and Feed Ingredients in Brazil: A Five-Year Survey

**DOI:** 10.3390/toxins14080552

**Published:** 2022-08-14

**Authors:** Gabriela L. Biscoto, Lauranne A. Salvato, Érika R. Alvarenga, Raul R. S. Dias, Guilherme R. G. Pinheiro, Mariana P. Rodrigues, Priscila N. Pinto, Rossimiriam P. Freitas, Kelly M. Keller

**Affiliations:** 1 Departamento de Medicina Veterinária Preventiva, Escola de Veterinária, Universidade Federal de Minas Gerais, Belo Horizonte 31270-901, MG, Brazil; 2 Departamento de Zootecnia, Escola de Veterinária, Universidade Federal de Minas Gerais, Belo Horizonte 31270-901, MG, Brazil; 3 Departamento de Química, Instituto de Ciências Exatas, Universidade Federal de Minas Gerais, Belo Horizonte 31270-901, MG, Brazil

**Keywords:** TMR, feed, maize, silages, co-occurrences, ELISA, aflatoxins, deoxynivalenol, zearalenone

## Abstract

Mycotoxins are toxic secondary metabolites produced by a variety of fungi, which when ingested can cause several deleterious effects to the health of humans and animals. In this work, the detection and quantification of six major mycotoxins (aflatoxins—AFLA, deoxynivalenol—DON, fumonisins—FUMO, ochratoxin A—OTA, T-2 toxin—T-2 and zearalenone—ZON) in 1749 samples of feed and feed ingredients for cattle, collected in Brazil between 2017 and 2021, was carried out using enzyme-linked immunosorbent assay (ELISA). In total, 97% of samples were contaminated with at least one mycotoxin, yet, very few samples exceeded the lowest European Union guidance values for cattle, and the estimated daily intake also showed a low risk for the animals. However, co-occurrences were widely observed, as 87% of samples contained two or more mycotoxins at the same time, and the presence of more than one mycotoxin at the same time in feed can lead to interactions. In conclusion, the contamination of feed and feed ingredients for cattle with mycotoxins in Brazil is very common. Hence, the monitoring of these mycotoxins is of significant importance for food safety.

## 1. Introduction

Mycotoxins are toxic secondary metabolites produced by a wide variety of fungi, especially species of the genera *Aspergillus*, *Penicillium*, and *Fusarium*. The deleterious effects of these compounds in the induction of carcinogenic, hepatotoxic, nephrotoxic, estrogenic, and mutagenic processes are recognized. The main mycotoxins found in animal feed are aflatoxins (AFLA), deoxynivalenol (DON), fumonisins (FUMO), ochratoxin A (OTA), T-2 toxin (T-2), and zearalenone (ZON) [1,2,3].

It is estimated that 25–50% of all commodities produced in the world are somehow contaminated with mycotoxins, with a significant impact on human and animal health. Ingestion of food and feed contaminated with high doses of mycotoxins promotes acute problems, which can lead to death. On the other hand, chronic intoxications, developed from the ingestion of food or feed contaminated with moderate doses of mycotoxins for prolonged periods, trigger changes, such as reduced growth and weight gain, decreased immunity, and greater susceptibility to diseases [4].

The global economic impact of mycotoxins on agriculture is difficult to measure, since it involves expenses with the loss of animals, veterinary care, reduced productivity of livestock and reduced grain supply. In this aspect, analyses of economic losses generated by mycotoxin contamination have been conducted individually for each chain involved. The US Food and Drug Administration (FDA) estimates that the annual losses in the United States in the corn, soybeans and peanuts crops are at US$932 million and another US$500 million are invested in research and monitoring of these fungal metabolites, resulting in a total loss of approximately US$1.5 billion annually [2]. In a more recent study, it was estimated that contamination by aflatoxins could cause losses to the corn industry of between US$52 million and US$1.7 billion annually to the USA alone, highlighting the importance monitoring and mycotoxin prevention and control measures to avoid losses resulting from contamination [5].

Another problem that is very frequently observed in feed are co-occurrences. Considering that many mycotoxigenic fungi can produce more than one mycotoxin and that several fungi species are found at the same time in raw materials used in animal feed, the study of the occurrence of a single mycotoxin provides incomplete information about the risk associated with the respective feed. When more than one mycotoxin is present in food or feed, three types of interactions can occur: synergistic, additive, or antagonistic (i.e., greater than, equal to or lower than the summed effects of the individual mycotoxins). Co-occurrences can cause great losses, as synergistic effects are often observed [6,7,8,9].

Several analytical methods can be used for the detection and quantification of mycotoxins in feed, with chromatographic techniques and immunochemical methods being the most used [10]. The ELISA (enzyme-linked immunosorbent assay) method for mycotoxin analysis has been available for over a decade and is currently a widely used immunoassay for the determination of mycotoxins. ELISA is an immunochemical technique considered to be fast and easy to perform and that does not require laborious sample preparation. For these reasons, it is widely used for the detection and quantification of mycotoxins in different matrices. Nowadays, several specific ELISA kits for the detection of numerous mycotoxins are commercially available [11,12,13,14,15,16].

In Brazil, cattle breeding has been present since the beginning of colonization and occupies a prominent role worldwide in relation to the production of both meat and milk, both activities being of great importance to the country since they represent a significant impact on the economy [17]. Beef and dairy cattle breeding must be constantly evolving, migrating to increasingly professional activities, aligned with the precepts of animal welfare and health safety. In addition, attention should also be paid to animal nutrition, since considerable amounts are spent on cattle nutrition, and feed contamination by toxins or microorganisms can be very harmful both for the health of animals and financially for producers, reducing the production of milk and meat, and consequently the exportation and consumption [18]. Therefore, mycotoxins must be monitored in cattle feed to avoid diseases and losses due to contamination.

Because of the potential effects on animal health resulting from the ingestion of feed contaminated with mycotoxins, several countries have legislations that establishes guidance values for mycotoxins in order to control feed contamination and reduce the economic losses resulting from this contamination. Brazil does not have regulations for mycotoxins in animal feed, and therefore European Union legislation is widely used as a parameter, as it is quite complete. Legislated mycotoxins by the European Union are aflatoxin B_1_, deoxynivalenol, fumonisins B_1_ + B_2_, ochratoxin A, T-2 toxin + HT-2 toxin, and zearalenone [19,20,21]. European Union legislation was used as a parameter for the selection of mycotoxins analyzed in this study, which were: total aflatoxins, deoxynivalenol, fumonisins B_1_ + B_2_, ochratoxin A, T-2 toxin and zearalenone. For aflatoxins analysis, total aflatoxins were analyzed and not just aflatoxin B_1_ (AFB_1_), as the ELISA kit used does not allow the exclusive quantification of AFB_1_, being only the quantification total aflatoxins possible. For T-2 + HT-2 toxins only the quantification of T-2 was possible since the ELISA kit available at the time of analysis allowed the quantification of only T-2 and not HT-2.

This study aimed to determine the occurrences and co-occurrences of mycotoxins in samples of feed and feed ingredients from Brazil between the years 2017 and 2021.

## 2. Results and Discussion

### 2.1. Mycotoxins Occurrence per Sample Type

The median values of positive samples, 1st quartile of positive samples, 3rd quartile of positive samples and maximum values, in addition to the number of analyzed and positive samples for each toxin (AFLA, DON, FUMO, OTA, T-2 and ZON) were determined for all samples together and for some sample types individually, namely TMR (Total Mixed Ration), silages, maize/maize products, finished feed, and other samples (Table 1).

In the analysis of all samples, it was observed that 97% of samples were contaminated with at least one of the studied mycotoxins. The most frequently found mycotoxin was DON, with a contamination frequency of 67.8%, followed by ZON and AFLA (with 62.5% and 58.6% of contaminated samples, respectively). For FUMO, OTA and T-2, the contamination rates were 40.8%, 38.8% and 28.6%, respectively (Table 1). These results show the high frequency of mycotoxin contamination in animal feed in Brazil, as has been observed by other authors in various parts of the world [6,22,23,24].

Still considering all samples, the median values calculated for the samples that showed a positive result (results greater than the LoD values) were: 4.0 µg/kg, 446.74 µg/kg, 940.0 µg/kg, 8.94 µg/kg, 27.12 µg/kg, 53.30 µg/kg for AFLA, DON, FUMO, OTA, T-2 and ZON, respectively (Table 1).

The maximum value found for AFLA was 266.60 µg/kg in a peanut meal sample from the state of São Paulo. The maximum DON value was 4969.06 µg/kg in a TMR sample from the state of Goiás. For FUMO, the highest value found was 31,420.00 µg/kg in a maize sample from Paraná. The highest OTA contamination value was 95.15 µg/kg in a maize silage sample from the state of Minas Gerais; 2959.06 µg/kg was the maximum found for T-2 in a citrus pulp sample from Minas Gerais. For ZON, the maximum value found was 2503.86 µg/kg in a TMR sample also from Minas Gerais.

In addition, for samples with a positive result for each toxin, the number and percentage of samples whose contamination exceeded the lowest guidance value recommended for each toxin by the European Union (EU) legislation was calculated (Table 2). For AFB_1_, DON, FUMO and ZON the lowest guidance values stablished for cattle feed were considered (5 µg/kg for AFLA; 2000 µg/ kg for DON; 20,000 µg/kg for FUMO and 500 µg/kg for ZON) [19,20]. For OTA and T-2 + HT-2, the reference value for cereals and cereal products was used for OTA (250 µg/kg) and reference values for compound feed were used for T-2 (250 µg/kg) [20,21]. A total of 36.5% of samples were contaminated with more than 5 µg/kg of total aflatoxins (360 samples), 5.6% (59 samples) contained more than 2000 µg/kg of DON, 2.1% (22 samples) were contaminated with more than 500 µg/kg of ZON, for T-2 six samples were contaminated with more than 250 µg/kg (1.4%), for FUMO only two samples showed contamination values above 20,000 µg/kg (0.3%), and for OTA none of the analyzed samples contained more than 250 µg/kg. It is important to highlight that for AFLA, total aflatoxins were analyzed, not only AFB_1_. This is the case because an immunochromatographic method (ELISA) was used, and with this type of method there is a percentage of cross-reactivity between similar molecules, with the quantification of only AFB_1_ not being possible. For T-2 + HT-2 only T-2 was quantified, since there was no T-2 + HT-2 ELISA kit available at the time of analysis.

It is also important to highlight that since only T-2 was quantified, and not HT-2, the number of samples exceeding 250 µg/kg is probably higher than the number observed. Moreover, since HT-2 was not considered in this study, it is also not possible to ensure that samples considered below the T-2 + HT-2 limit (250 µg/kg) were in fact below this limit.

These results indicate that most samples from Brazil are contaminated with levels of mycotoxins below the lowest guidance values recommended by the European Union, however, in the global analysis of feed several other factors need to be taken into consideration, such as breed, sex, environment, and the nutritional and immunological status of the animals [6], in addition to the presence of multiple mycotoxins in the same sample, which can worsen the effects observed due to synergistic interactions.

In TMR (total mixed ration) samples, high percentages of contamination by ZON, DON and AFLA were observed (77.5%, 70.3%, and 65.7%, respectively), higher percentages than those observed for all the samples together. As TMRs are prepared from several different commodities, the same sample contains several sources of contamination by different mycotoxins, resulting in the high rates of contamination observed. Still for the TMR samples, the median values of the positive samples were 3.86 µg/kg for AFLA, 430.00 µg/kg for DON, 613.84 µg/kg for FUMO, 8.31 µg/ kg for OTA, 23.40 µg/kg for T-2 and 55.23 µg/kg for ZON (Table 1). Twaruzek et al. (2021) performed a study in Poland, and all analyzed TMR samples contained ZON and DON. However, AFLA was not present in any of the samples [25].

In silage samples, the most common mycotoxins were OTA, ZON, AFLA, and DON (61.4%, 61.2%, 55.1% and 52.5%, respectively). The medians of the positive samples were 4.00 µg/kg, 300.00 µg/kg, 820.00 µg/kg, 23.30 µg/kg, 35.98 µg/kg, and 43.86 µg/kg for AFLA, DON, FUMO, OTA, T-2, and ZON, respectively (Table 1).

As the silage process is mainly carried out by farmers, great differences in the quality of preservation of these silages are observed, leading to the presence of different fungi and a varied spectrum of mycotoxins [26].

Scientific publications on the contamination of silages by mycotoxins are quite scarce when compared to those that assess contamination in cereals. However, the contribution of silages to the total intake of mycotoxins can be significant, as forages are the main dry matter component of bovine diets [26,27]. In a study carried out by Driehuis et al. (2008) in the Netherlands, a high prevalence of DON (72%) and ZON (49%) was observed in maize silage samples. Wheat and grass silages were less contaminated, ZON was present in 6% of grass silage samples, and 10% of wheat silages contained DON. AFLA and OTA were not detected in any of the silages studied [28].

In the maize/maize products samples, FUMO was the predominant mycotoxin, with 85.9% of contaminated samples, followed by DON, which was found in 61.8% of the analyzed maize samples. These results agree with the findings of Streit et al. (2013), who also observed a high prevalence of FUMO and DON in maize samples from all over the world [22]. The 1st quartile, median and 3rd quartile values found for FUMO in maize samples were 955.00 µg/kg, 1695.02 µg/kg and 3245.99, respectively, these being the highest values found for FUMO among all analyzed sample types (Table 1). It is known that FUMO is a very commonly found mycotoxin in maize, since its main producer (*Fusarium verticillioides*) is known to be a maize phytopathogen [29].

In the finished feed samples, the most frequent mycotoxins were DON (87.9% of contamination), FUMO (73.6%), and ZON (55.8%). For this sample type, the median values of positive samples were 6.22 µg/kg for AFLA, 690.00 µg/kg for DON, 970.00 µg/kg for FUMO, 6.34 µg/kg for OTA, 24.11 µg/kg for T-2, and 43.40 µg/kg for ZON (Table 1). The large amount of mycotoxin-contaminated finished feed samples is not surprising, since finished feed, like TMR samples, are a mixture of different commodities and therefore also contain a mixture of mycotoxins from these commodities. In addition, maize and maize products are often added in large amounts to finished feeds, and consequently both maize and finished feeds have a high prevalence of fumonisins [24].

Among the other samples, which include soybean, cottonseed, peanut meal, citrus pulp, barley, wheat and sorghum, the main mycotoxins found were DON (present in 70.2% of samples), AFLA (present in 64.6% of samples) and ZON (present in 58.8% of samples). In addition, for these samples, the median values of the positive samples were 8.37 µg/kg, 688.07 µg/kg, 540.00 µg/kg, 4.15 µg/kg, 27.12 µg/kg and 66.41 µg/kg for AFLA, DON, FUMO OTA, T-2, and ZON, respectively (Table 1).

Among the other samples, the peanut meal samples stand out. The presence of AFLA in peanuts is a major problem in Brazil [30], and in this study it was observed that all 37 peanut meals samples analyzed were contaminated with AFLA, with a median of 73.38 µg/kg.

Although low levels of contamination were observed in most samples, the monitoring of these mycotoxins is of significant importance due to the possibility of chronic intoxication, especially in young animals, and the possibility of adverse effects due to co-occurrences. In addition, another aggravating factor is the carry-over of mycotoxins and their metabolites to meat and milk. When animals are fed with diets contaminated with mycotoxins, these mycotoxins are subjected to enzymatic and microbial transformations that lead to the formation of metabolites in the intestine. The resulting metabolites can then be absorbed into the animal’s bloodstream and subsequently excreted in the urine and/or feces. However, toxins and their metabolites that are not excreted from the body remain in organs, muscles, and milk, and can then be ingested by humans [31,32,33,34].

Furthermore, studies evaluating the presence of mycotoxins in feeds intended for cattle in Brazil are scarce, and the high frequency of contamination observed in this study highlights the need for frequent monitoring of these feeds to ensure its safety and animal’s welfare. Another aggravating factor in Brazil is the lack of specific legislation for mycotoxin contamination in animal feed, which puts the health of animals at risk.

### 2.2. Mycotoxins Occurrence per Year

**Figure 1:**
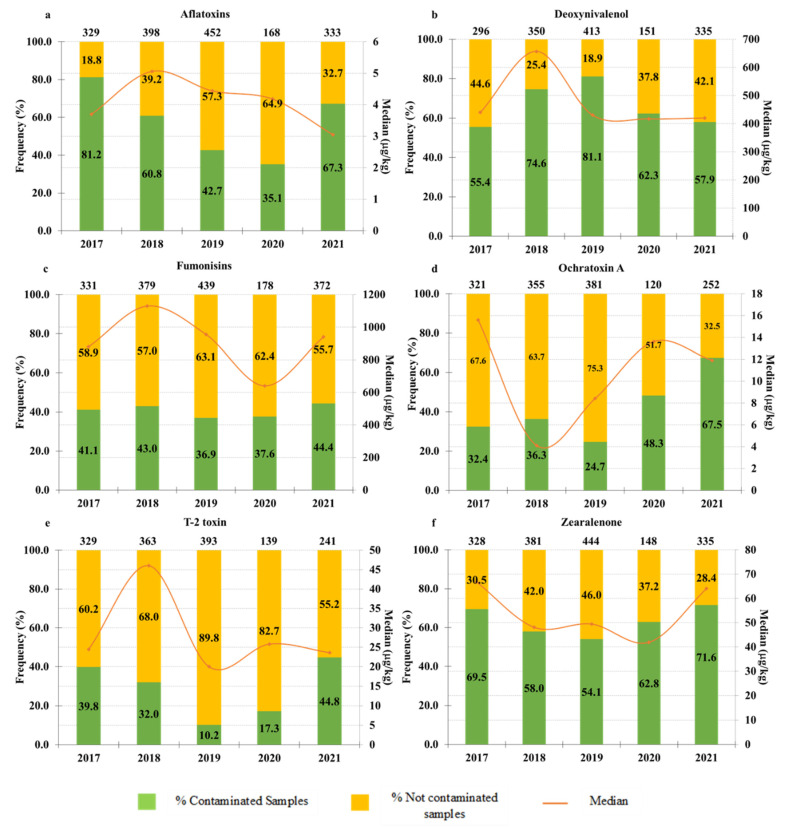
Percentage of contaminated and not contaminated samples and median of positive samples for (**a**) AFLA, (**b**) DON, (**c**) FUMO, (**d**) OTA, (**e**) T-2 and (**f**) ZON for each studied year (2017–2021). Numbers above the bars represent the number of analyzed samples each year.

The percentage of contaminated and not contaminated samples and the median of positive samples were determined for each toxin (AFLA, DON, FUMO OTA, T-2 and ZON) in each of the years studied (2017 to 2021) (Figure 1).

For aflatoxins, the highest percentage of contamination occurred in 2017 (81.2%) and the lowest in 2020 (35.1%). Regarding the median values, there was little variation over the years—3.7 µg/kg, 5.06 µg/kg, 4.44 µg/kg, 4.18 µg/kg and 3.05 µg/kg for the years 2017, 2018, 2019, 2020 and 2021, respectively (Figure 1). Aflatoxins are very common in Brazilian commodities, since the country has favorable climatic conditions for the growth of the main fungi producing this toxin (*Aspergillus flavus* and *A. parasiticus*). These fungi have a worldwide distribution, but occupy mainly regions of tropical and subtropical climate, growing at high temperatures and low water activity [35].

For deoxynivalenol, all years showed percentages of contamination above 50%, with 2019 being the year with the highest DON contamination (81.1%) and 2017 the year with the lowest percentage of contaminated samples (55.4%). Regarding the median, there was little variation between the years 2017, 2019, 2020, and 2021. Only in 2018 was there an increase in this parameter (656.67 µg/kg) (Figure 1).

For fumonisins, in all years the percentages of contamination were lower than 50%, with little variation between years. On the other hand, the median ranged between 640.0 µg/kg in 2020 and 1129.66 in 2018 (Figure 1).

For ochratoxin A, the highest percentage of contamination was observed in 2021 (67.5%) and the lowest in 2019 (24.7%). In relation to the median, there was also great variation, with the highest value observed in 2017 (15.59 µg/kg) and the lowest in 2018 (4.11 µg/kg) (Figure 1).

Regarding T-2 toxin, the year with the highest percentage of contaminated samples was 2021 with 44.8% of contamination, while the year with the lowest percentage of contaminated samples was 2019 with only 10.2% of contamination. The median of this toxin was between 20.0 µg/kg (in 2019) and 46.0 µg/kg (in 2018) (Figure 1). Among the mycotoxins analyzed in this study, T-2 was the least prevalent, but the prevalence of T-2 is quite variable with some countries showing high frequencies of this toxin, while in others the contamination is low. A study carried out in Poland between 2015 and 2020 observed that 88.4% of the analyzed samples contained T-2, with a maximum value found of 898 μg/kg [25]. In another study carried out in Slovakia, T-2 was the most frequently found mycotoxin, present in 90% of the analyzed samples, but in low concentrations (average of 13 µg/kg) [36]. On the other hand, Drakopoulos et al. (2021) analyzed 253 barley samples originating in Switzerland and found only 8% of T-2 contamination, with a median of 5.1 μg/kg [37].

For zearalenone, as well as for DON, the contamination was above 50% in all years, with 71.6% of samples contaminated in 2021. The median varied little over the years, being 41.97 µg/kg in 2020 and 66.74 µg/kg in 2017 (Figure 1).

There are many factors that can influence mycotoxin contamination, with climate being a very important one [38,39]. Thus, changes in temperature, precipitation, relative humidity, and atmospheric concentration of CO_2_ can alter the ability of fungi to produce mycotoxins, causing annual increases or reductions in the relative risk of mycotoxin contamination both in the field and post-harvest [35,39].

Fungi of the genus *Fusarium* spp. (DON, FUMO, T-2 and ZON producers) adapt easily to a wide variety of habitats, having worldwide distribution. These fungi are important plant pathogens and, the occurrence of different mycotoxins in the field is related to the geographic location of the crop and the meteorological conditions. Therefore, mycotoxin prevention and control techniques, such as good agricultural practices and good practices during the manufacture, handling, storage, processing, transportation and distribution of food and feed, play an important role in minimizing mycotoxin contamination [35].

**Figure 2:**
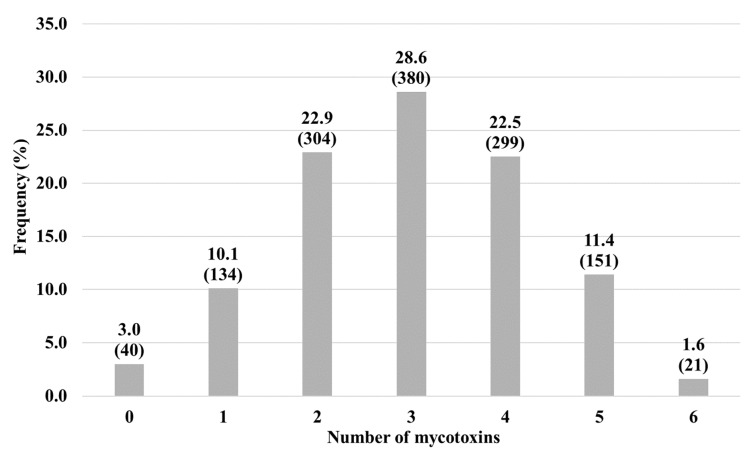
Relative frequency of samples contaminated with zero, one, two, three, four, five or six mycotoxins at the same time. Numbers in parentheses are the total sample number.

In addition to climatic variations, the annual variations observed in Figure 2 may be due to other factors, such as: the timing of harvest and post-harvest handling and storage. In addition, annual variations can also be due to variations in the sample types analyzed in each year, i.e., the proportion of each sample type (TMR, silages, maize/maize products, finished feed and other samples) analyzed in each year was variable, and as noted in Section 2.1 contaminations are different in different sample types.

### 2.3. Mycotoxin Co-Occurrences

For the analysis of co-occurrences, 1329 samples that were tested for all six mycotoxins (AFLA, DON, FUMO, OTA, T-2 and ZON) were used. In total, 87% of samples were contaminated with two or more mycotoxins at the same time, with 22.9% being contaminated with two mycotoxins, 28.6% with three, 22.5% with four, 11.4 with five, and 1.6% contained six mycotoxins at the same time (Figure 2). Other studies also report high frequencies of co-occurrences in animal feed in various parts of the world [23,24,40,41].

The fraction of samples contaminated with each possible combination of two mycotoxins was also calculated (Table 3). The three most frequent combinations were DON + ZON, AFLA + DON, and AFLA + ZON, which were present in 45.2%, 42.1%, and 41.5% of samples, respectively. The most frequent mycotoxins in this study were DON, ZON, and AFLA, so the most frequent co-occurrences also involve these toxins. Furthermore, the most observed combination was DON + ZON, which is expected since these toxins are mainly produced by the same fungi *(Fusarium graminearum* and *Fusarium culmorum*), and co-contamination with these mycotoxins is frequently observed and has been described in other studies [23,24,25,28].

Although scientific literature offers a wide range of information on the individual effects of mycotoxins in various animal species, studies on the combined toxic effects of these toxins in vivo are limited, and therefore the health risk associated with the exposure to a combination of mycotoxins is incomplete. In field outbreaks, naturally contaminated feeds may contain multiple mycotoxins, and lower rates of contamination may be associated with more serious effects due to the combined action of mycotoxins. This explains the differences observed in the effects described in the scientific literature and in the cases observed in field outbreaks [6,42,43]. Furthermore, the risk of exposure to multi-mycotoxins in ruminants is even greater than in other animals (such as pigs and poultry), since the diet of these animals is more varied, containing several possible sources of contamination [27].

Studies on the toxic effects of mycotoxin combinations have already been carried out in some animal species, but research on these effects in cattle are still lacking. The presence of DON + ZON (which was the most frequent combination of toxins in this study) in feed may result in additive, synergistic, or antagonistic interactions depending on the dose used and the animal species studied. Synergistic and additive effects have already been described on immunological parameters in mice and pigs [44,45], and in the brain and kidney of mice [46,47]. Antagonistic effects have also been described on immunological parameters of mice [48,49].

Existing legislations have also been established based on toxicological studies that consider exposure to only one mycotoxin and not to mycotoxin mixtures [8]. However, the results of this study indicate that the co-occurrence of mycotoxins in feed and feed ingredients used for cattle feeding in Brazil, and the consequent exposure of animals to several mycotoxins at the same time is the rule and not the exception, and it is very important to consider the combined toxic effects of these mycotoxins.

### 2.4. Estimated Daily Intake

When carrying out a risk assessment, one should consider not only the absolute concentrations of mycotoxins in feed (expressed in µg of mycotoxin/kg of feed), but also the daily intake of these toxins by the animals (expressed in µg of mycotoxin/kg body weight/day) [50]. For this estimation of the daily exposure of beef and dairy cattle to mycotoxins, only the TMR samples were used, and the body weight and food intake highlighted in Section 4.5 were considered.

**Figure 3:**
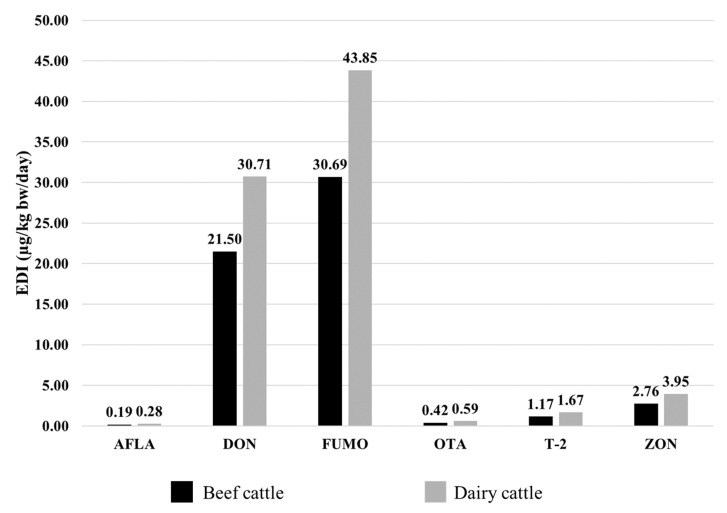
Estimated daily intake (EDI) of mycotoxins in TMR for beef cattle (black bars) and dairy cattle (grey bars).

The highest EDI value was found for FUMO (30.69 µg/kg bw/day for beef cattle and 43.85 µg/kg bw/day for dairy cattle) followed by DON with an EDI of 21.50 µg/kg bw/day beef cattle and 30.71 µg/kg bw/day for dairy cattle. For all other mycotoxins, the EDIs found were low, with the lowest observed for AFLA (0.19 µg/kg bw/day for beef cattle and 0.28 µg/kg bw/day for dairy cattle) (Figure 3). The values found do not represent a risk for the animals, but attention must be paid to co-contaminated samples, because in these samples the EDI increases due to the presence of several mycotoxins in the same feed.

## 3. Conclusions

Few studies evaluate mycotoxin contamination in animal feed in Brazil, and the high frequencies of contamination observed in this study emphasize the importance of improving mycotoxin control in the country, as well as the need to adopt regulations for mycotoxins in animal feed in Brazil. In this study, most of the analyzed samples showed contamination below the lowest guidance values established by the European Union, but co-occurrences are widely observed, and knowledge about these co-occurrences remains scarce. Meanwhile, more data on these interactions and their possible effects on cattle are needed.

## 4. Materials and Methods

### 4.1. Samples and Sampling

**Figure 4:**
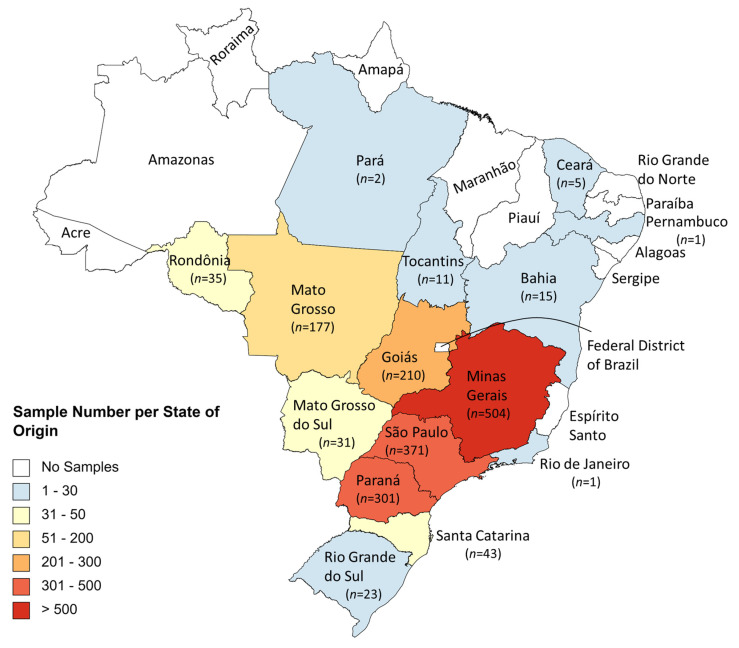
Number of samples per state of origin. The number of analyzed samples for each state are given (*n*).

A total of 1749 samples of feed and feed ingredients for cattle were taken from different Brazilian states (Figure 4) between January 2017 and December 2021. Samples were classified into five groups, namely: total mixed ration (TMR) (639 samples), silages (252 samples), maize/maize products (244 samples), finished feed (166 samples) and other samples (which include soybean, cottonseed, peanut meal, citrus pulp, barley, wheat and sorghum) (405 samples). For 43 samples, the sample type was not informed.

Samples were sent to the laboratory by the producers, and therefore the sampling was not evaluated. However, producers were instructed on the correct form of sampling [51].

### 4.2. Chemicals and Reagents

Methanol (MeOH) ACS reagent ≥ 99.5% wes purchased from Neon Comercial Reagentes Analíticos (São Paulo, Brazil) and water was obtained from a 5-stage reverse osmosis system (Hydronix Water Technology, Chinohills, CA, USA). Certified Rainin™ LTS pipettes (Mettler-Toledo, Columbus, OH, USA) and laboratory glassware were also used for all the analysis.

### 4.3. Mycotoxins Extraction

For the extraction of mycotoxins, two extracts were prepared: one for the quantification of deoxynivalenol (DON) and the other for the quantification of the other toxins (aflatoxins—AFLA, fumonisins—FUMO, ochratoxin A—OTA, T-2 toxin—T-2 and zearalenone—ZON). For the preparation of the extracts, samples were completely grounded (Ninja^®^ Nutri-Blender with Auto-iQ^®^, SharkNinja, Needhan, MA, USA) and homogenized, then, two 20 g fractions were weighed (Balance BL3200H, Shimadzu, Kioto, Japan). In one of the fractions, 100 mL of distilled water was added (for DON quantification) and in the other 100 mL of a methanol:water solution (70:30 *v/v*) was added (for the quantification of AFLA, FUMO, OTA, T-2 and ZON). Then, samples added to distilled water or methanol/water were stirred at 150 rpm (revolutions per minute) at 25 °C in an orbital shaker (CERTOMAT^®^ BS-1, Sartorius, Goettingen, Germany) for one hour, after which the samples were filtered using Whatman #1 filter. The filtrate was directly used for the quantification of AFLA, OTA, and T-2. For DON, FUMO and ZON an additional dilution is required. Dilutions are 1:4 in distilled water for DON, 1:20 in distilled water for FUMO and 1:5 in 70% methanol for ZON. The filtrates and diluted filtrates were then used for the quantification of mycotoxins.

### 4.4. Mycotoxins Analysis

All mycotoxin analyses were performed using the enzyme-linked immunosorbent assay (ELISA). This method was chosen because it is considered to be of high yield and requires fewer extract purification procedures compared to other conventional methods, in addition to not requiring expensive equipment that is often not available. The ELISA method is totally quantitative, fast, simple, and sensitive, therefor it can be used for the detection of mycotoxins in various foods and feeds [11,12]. In this study the following mycotoxins were investigated: total aflatoxins (B_1_, B_2_, G_1_ and G_2_) (AFLA), deoxynivalenol (DON), total fumonisins (B_1_, B_2_ and B_3_) (FUMO), ochratoxin A (OTA), T-2 toxin (T-2) and zearalenone (ZON). AgraQuant^®^ ELISA kits produced by Romer Labs Inc. (Getzerstorf, Austria) were used and analysis were performed following the manufacturer’s instructions. The following ELISA kits were used: AgraQuant^®^ Total Aflatoxin 1/20, AgraQuant^®^ Deoxyniva-lenol 0.25/0.5, AgraQuant^®^ Fumonisin 0.25/5.0, AgraQuant^®^ Ochratoxin 2/40, AgraQuant^®^ T-2 Toxin 20/500 and AgraQuant^®^ Zearalenone Plus 25/1000 (Romer Labs Inc., Getzerstorf, Austria). These kits are validated by the manufacturer on over 50 matrices, including complex matrices such as maize silage, DDGs, feed, peanuts etc. [52,53,54]. In addition, other authors also performed validation studies using AgraQuant^®^ kits and obtained good performance results, comparable to HPLC methods [55,56].

The method’s limits of detection (LoD) and limits of quantification (LoQ) are shown in Table 4. Samples with results lower than the LoD values were considered not contaminated, while samples with results between the LoD and LoQ were assigned the LoQ values.

To perform the tests, first, the samples were mixed with a mycotoxin-enzyme conjugate. Then, the mixture of sample + conjugate was transferred to antibody-coated microwells. After an incubation period, the wells were washed, an enzyme substrate was added, and blue color developed. The color intensity is inversely proportional to the concentration of mycotoxin in the sample. The reaction was then stopped with a stop solution, which changes the color from blue to yellow. Finally, the color intensity of each well was measured optically using an ELISA reader (Stat Fax 303 Plus Microstrip Reader, Awareness Technologies, Westport, CT, USA) with a 450 nm absorbance filter and a 630 nm differential filter. The optical densities (OD) of the samples were compared to the OD of standards through a linear regression, thus obtaining the concentration of mycotoxin present in each sample. Only calibration curves with R^2^ greater than 0.99 were considered. Analyses were performed in duplicate for each sample.

It is also important to highlight that in order to check and guarantee the efficiency of the analyzis performed, the laboratory regularly participates in the Interlaboratory Proficiency Tests developed by Romer Labs^®^ (Romer Labs^®^ Check-Sample-Survey Programme) using the ELISA technique and obtaining satisfactory results.

### 4.5. Descriptive Analysis

Descriptive data analysis was performed using the Microsoft 365^®^ Excel (2021) program. The total number of analyzed samples for each mycotoxin was calculated, in addition to the absolute and relative frequencies of contaminated samples. The 1st quartile of positive samples, median of positive samples, 3rd quartile of positive samples, and maximum values for each mycotoxin were also calculated. Analyses were performed per sample type and per year.

In addition, the quantitative values of each mycotoxin were compared with the lowest guidance values recommended for each toxin by the European Union (EU) legislation for cattle feed. For AFB_1_, the guidance value used was 5 µg/kg, for DON 2000 µg/kg, for FUMO 20,000 µg/kg, and ZON 500 µg/kg. For OTA and T-2 + HT-2, since there are no guidance values established specifically for cattle for these toxins, a reference value for cereals and cereal products was used for OTA (250 µg/kg) and a reference value for compound feed was used for T-2 + HT-2 (250 µg/kg) [19,20,21].

For co-occurrences, the number and relative frequency of samples contaminated with zero, one, two, three, four, five, or six mycotoxins at the same time were determined. In addition, the number of samples that contained each of the two-by-two combinations of mycotoxins and the relative frequencies of these combinations were also calculated.

### 4.6. Estimated Daily Intake (EDI)

The estimated daily intake (EDI) calculation was performed separately for dairy cattle and beef cattle. It was considered that a dairy cow consumes an average of 50 kg of TMR per day and weighs 700 kg. For beef cattle, it was considered that an animal weighs on average 600 kg and consumes 30 kg of TMR. The EDI (in µg mycotoxins/kg body weight/day) was calculated for each mycotoxin using the following Equation (1) [25,57]:

EDI = (Tc × Fi)/M(1)

where 

Tc: mycotoxin concentration (median value for TMR was used; µg of mycotoxin/kg of food);

Fi: feed intake (30 kg for beef cattle and 50 kg for dairy cattle);

M: body weight (600 kg for beef cattle and 700 kg for dairy cattle).

## Figures and Tables

**Table 1:** Contamination rates in different sample types.

Mycotoxins	*n* ^1^	Positive Samples		1st Quartile (µg/kg) ^2^	Median (µg/kg) ^3^	3rd Quartile (µg/kg) ^4^	Maximum (µg/kg) ^5^
		*n* ^1^	%				
	All Samples						
AFLA	1680	985	58.6	2.55	4.00	7.08	266.60
DON	1545	1048	67.8	291.67	446.74	960.00	4969.06
FUMO	1699	693	40.8	460.00	940.00	1860.00	31,420.00
OTA	1429	555	38.8	4.00	8.94	20.75	95.15
T-2	1465	419	28.6	20.00	27.12	52.19	2959.06
ZON	1636	1022	62.5	33.94	53.30	89.92	2503.86
	TMR						
AFLA	626	411	65.7	2.43	3.86	4.92	61.54
DON	600	422	70.3	290.00	430.00	1086.22	4969.06
FUMO	633	249	39.3	370.00	613.84	1190.26	17,490.00
OTA	537	260	48.4	4.09	8.31	15.75	87.82
T-2	540	169	31.3	20.00	23.40	54.00	86.32
ZON	632	490	77.5	36.84	55.23	86.32	2503.86
	Silages						
AFLA	236	130	55.1	2.51	4.00	4.59	14.62
DON	219	115	52.5	250.00	300.00	415.58	2747.80
FUMO	242	63	26.0	380.00	820.00	1710.00	17,732.00
OTA	184	113	61.4	9.75	23.30	45.69	95.15
T-2	210	44	21.0	20.00	35.98	62.55	132.23
ZON	237	145	61.2	30.93	43.86	67.07	1900.52
	Maize/Maize Products						
AFLA	223	94	42.2	2.32	4.00	5.43	82.13
DON	212	131	61.8	260.00	323.14	450.18	1390.00
FUMO	241	207	85.9	955.00	1695.02	3245.99	31,420.00
OTA	189	72	38.1	4.09	13.52	31.16	86.79
T-2	195	36	18.5	20.00	41.10	57.15	106.58
ZON	207	65	31.4	26.53	45.75	102.27	1430.07
	Finished Feed						
AFLA	156	73	46.8	2.80	6.22	10.54	66.66
DON	141	124	87.9	415.51	690.00	1025.00	1980.26
FUMO	159	117	73.6	580.00	970.00	1574.69	7997.78
OTA	143	16	11.2	3.40	6.34	22.36	81.35
T-2	141	30	21.3	20.00	24.11	46.46	135.23
ZON	147	82	55.8	29.32	43.40	69.78	365.80
	Other Samples						
AFLA	398	257	64.6	3.82	8.37	17.80	266.60
DON	336	236	70.2	378.49	688.07	1255.00	4828.98
FUMO	382	41	10.7	359.59	540.00	1412.00	6791.16
OTA	341	81	23.8	3.24	4.15	8.94	78.00
T-2	345	126	36.5	20.00	27.12	46.38	2959.06
ZON	374	220	58.8	39.14	66.41	106.48	1450.13

^1^ Sample number. ^2^ 1st quartile of positive samples. ^3^ Median of positive samples. ^4^ 3rd quartile of positive samples. ^5^ Maximum values detected for each mycotoxin.

**Table 2:** Percentage of samples exceeding guidance values recommended for each toxin by the European Union (EU) legislation.

Mycotoxin	Lowest EU Guidance Value	*n* ^1^	% ^2^
AFLA	5 µg/kg	360	36.5
DON	2000 µg/kg	59	5.6
FUMO	20,000µg/kg	2	0.2
OTA	250 µg/kg	0	-
T-2	250 µg/kg	6	1.4
ZON	500 µg/kg	22	2.1

^1^ Number of samples above the lowest EU guidance values for each toxin. ^2^ Percentage of samples above the lowest EU guidance values for each toxin.

**Table 3:** Frequency of samples contaminated with each possible two-by-two combination of mycotoxins.

Mycotoxins Combination	Frequency of Contamination (%)
DON + ZON	45.2
AFLA + DON	42.1
AFLA + ZON	41.5
DON + FUMO	30.7
OTA + ZON	26.7
AFLA + FUMO	24.5
DON + OTA	23.5
FUMO + ZON	22.9
AFLA + OTA	20.9
AFLA + T-2	20.5
T-2 + ZON	20.2
DON + T-2	16.6
OTA + T-2	14.4
FUMO + OTA	14.1
FUMO + T-2	11.3

**Table 4:** Limits of detection (LoD) and limits of quantification (LoQ) for the six mycotoxins studied.

Mycotoxins	Limits of Detection (LoD) (µg/kg)	Limits of Quantification (LoQ) (µg/kg)
Total Aflatoxins	1.0	1.0
Deoxynivalenol	200.0	250.0
Fumonisins	200.0	250.0
Ochratoxin A	1.9	2.0
T-2 toxin	10.0	20.0
Zearalenone	20.0	25.0

## Data Availability

Not applicable.
